# Time-restricted feeding improves metabolic syndrome by activating thermogenesis in brown adipose tissue and reducing inflammatory markers

**DOI:** 10.3389/fimmu.2025.1501850

**Published:** 2025-01-24

**Authors:** Yueling Gong, Honghui Zhang, Jiang Feng, Li Ying, Mengmeng Ji, Shiyin Wei, Qiming Ma

**Affiliations:** ^1^ Department of General Surgery, First Affiliated Hospital of Gannan Medical University, Ganzhou, China; ^2^ Department of Traditional Chinese Medicine, Xiang’an Hospital of Xiamen University, Xiamen, Fujian, China; ^3^ Department of Neurosurgery, Affiliated Hospital of Youjiang Medical University for Nationalities, Baise, Guangxi, China; ^4^ Key Laboratory of Medical Research Basic Guarantee for Immune-Related Diseases Research of Guangxi (Cultivation), Guangxi, China

**Keywords:** time-restricted feeding, inflammation, metabolic syndrome, hepatic steatosis, treatment

## Abstract

**Background:**

Obesity and metabolic syndrome (MetS) have become increasingly significant global health issues. Time-restricted feeding (TRF), as a novel dietary intervention, has garnered attention in recent years. However, there is limited research focusing on the effects of TRF on energy expenditure and systemic low-grade inflammation. This study aims to investigate the impact of TRF on weight management, glucose metabolism, insulin resistance, and lipid metabolism in male C57BL/6J mice, particularly in the context of metabolic disorders induced by a high-fat diet (HFD).

**Methods:**

C57BL/6J mice were divided into two groups: a normal diet (ND) group and a high-fat diet (HFD) group. The study duration was 12 weeks. Key parameters observed included body weight, glucose tolerance (via glucose tolerance tests), insulin resistance (HOMA-IR), and insulin secretion under glucose stimulation. Additionally, liver tissue was subjected to Oil Red O staining to assess lipid accumulation, and white and brown adipose tissues were stained with hematoxylin and eosin (HE) to evaluate adipocyte size. The expression of hepatic lipogenesis-related genes (Srebp-c, Chrebp, Fasn, and Acc1) and thermogenic genes in brown adipose tissue (UCP1 and PGC-1α) were also measured. Furthermore, temperature changes in the interscapular brown adipose tissue (BAT) were monitored.

**Results:**

In the ND group: TRF improved insulin resistance and reduced circulating levels of the pro-inflammatory cytokine IL-6, with a slight reduction in body weight.In the HFD group: TRF significantly mitigated weight gain, improved glucose tolerance and insulin resistance, and enhanced insulin secretion under glucose stimulation. Additionally, TRF reduced hepatic steatosis by downregulating the expression of lipogenesis-related genes in the liver. TRF also increased thermogenesis by upregulating the expression of thermogenic genes (UCP1 and PGC-1α) in BAT, while lowering serum levels of pro-inflammatory cytokines IL-6 and TNF-α, though IL-1β levels remained unchanged.

**Conclusion:**

This study demonstrates that TRF can activate thermogenesis in brown adipose tissue and reduce inflammation maker, leading to an improvement in hepatic steatosis and a reduction in white adipose tissue accumulation. These findings suggest that TRF may be a promising intervention for mitigating metabolic disturbances associated with obesity and metabolic syndrome. The study provides mechanistic insights into the beneficial effects of TRF, highlighting its potential in modulating lipid metabolism and exerting anti-inflammatory effects.

## 1 Introduction

Metabolic syndrome (MetS) is a growing global concern, leading to increased attention within the medical community on the development of innovative therapies to combat its pathophysiological effects (1, 2). MetS is characterized by a cluster of metabolic abnormalities, including central obesity, insulin resistance, hypertriglyceridemia, hypercholesterolemia, hypertension, and reduced levels of high-density lipoprotein (HDL) cholesterol (3). It is also associated with several comorbidities, including a pro-inflammatory and pro-thrombotic state, metabolic-associated fatty liver disease (MAFLD), cholesterol gallstone disease, and reproductive disorders. MetS is recognized as a major risk factor for the global epidemics of type 2 diabetes mellitus (T2DM) and cardiovascular diseases in the 21st century (4). In the United States, the prevalence of MetS is estimated to be 10.18% (5). Approximately 35% of American adults have prediabetes, and 17.8% of them, without intervention, will progress to T2DM within five years (6). The global incidence of T2DM is approaching pandemic levels and is closely linked to the rising rates of obesity. By 2030, it is projected that over 39% of adults worldwide will be overweight or obese, with around 25% falling into the category of severe obesity, defined as a body mass index (BMI) ≥35 kg/m² (7, 8). Furthermore, according to the World Health Organization (WHO), approximately 17.9 million people die each year from cardiovascular diseases related to MetS, accounting for about one-third of all deaths. These statistics underscore the urgent need for strategies to prevent or treat MetS.

While recent advances in obesity therapies have been promising, there is also a growing interest in improving dietary patterns through various nutritional interventions. Time-restricted feeding (TRF), a form of intermittent fasting, has gained significant attention, with the 16:8 model (where food intake is limited to an 8-hour window each day) being a popular subject of research. In this model, food can be consumed during the 8-hour window, with only water allowed during the remaining 16 hours (9). Recent clinical studies have shown that early TRF, starting at 06:00 AM, can further improve metabolic parameters such as fasting glucose and insulin sensitivity (10, 11). TRF has been highlighted by health professionals as an effective dietary approach for weight management, cardiovascular health, and reducing oxidative stress (12). This fasting regimen has been shown to induce favorable metabolic changes, including improved glycemic control, reduced glycogen stores, increased fatty acid and ketone body release, decreased leptin levels, and elevated adiponectin levels (13–16). In overweight or obese adults, fasting has been reported to lead to reductions in BMI, body weight, waist circumference, and fat mass (17–21).

Despite these benefits, much of the research to date has focused on the reduction in caloric intake as the primary mechanism behind these positive outcomes, with less attention given to the potential increase in energy expenditure. Recent studies have found that a 5:2 TRF model, which involves five days of ad libitum feeding and two days of fasting, can increase exercise-induced energy expenditure (22). Although this model differs from typical healthy eating patterns, it suggests that TRF (16:8) may also enhance energy expenditure. Additionally, some studies have noted that regular fasting can reduce circulating inflammatory markers, which could contribute to improved health outcomes.

There are various TRF patterns, including 4:3, 5:2, and 16:8. The 16:8 or 16 + 8 TRF model is the most popular, but there is limited research focusing on fat thermogenesis and systemic inflammation regulation (23, 24). Therefore, the primary aim of this study is to investigate the effects of the 16 + 8 TRF model on fat thermogenesis and inflammation maker. Using mice as the model organism, we will focus on how fasting regulates thermogenesis in brown adipose tissue (BAT) and the inflammatory maker response, thus offering new insights into TRF as a potential therapeutic strategy for MetS.

## 2 Materials and methods

### 2.1 Animal experiment and study design

A total of 56 male C57BL/6J mice, aged 6–8 weeks, were purchased from Jackson Laboratory (Bar Harbor, Maine) for this study. All mice were housed in a clean and ventilated environment and fed with standard diet (4–5% calories, SPFW01, SCBS, China) and high-fat diet (60% calories, D12492, research diet, USA). During the initial 2-week acclimation period, all animals were trained under controlled conditions: 12 hours of light and 12 hours of darkness, with ad libitum access to food and water. After two weeks, the animals were randomly assigned to one of two groups for 12 weeks: (i) a control group with free access to food and water during the 12-hour light and 12-hour dark cycles (Control), and (ii) a time-restricted feeding (TRF) group, where food was restricted to an 8-hour window (8:00 AM to 4:00 PM) under the same light/dark cycle. The feeding times were controlled by trained personnel. The experimental protocol was approved by the Ethics Committee of the First Affiliated Hospital of Gannan Medical University. After completing the experiments, tissue samples were collected and processed for biological analysis in an environmentally safe manner.

### 2.2 Assessment of glucose tolerance, insulin secretion, and insulin sensitivity

At 8:00 AM, glucose tolerance and insulin secretion were assessed in all mice after a 16-hour fasting period. For the glucose tolerance test, the mice received an intraperitoneal injection of 20% glucose (2 g/kg body weight). Blood glucose levels were measured at baseline and 15, 30, 45, 60, and 120 minutes post-injection. Plasma samples were collected at baseline and at 2.5, 5, and 15 minutes post-injection to assess insulin levels. For the insulin tolerance test, insulin sensitivity was assessed after a 6-hour fast at 10:00 PM. The mice were injected intraperitoneally with insulin (0.55 mU/g body weight), and blood glucose levels were measured at baseline and 15, 30, 45, 60, and 120 minutes post-injection.

### 2.3 RNA isolation and quantitative RT-PCR

Total RNA was extracted from freshly collected mouse liver and adipose tissues using the RNeasy Mini Kit (Qiagen, Hilden, Germany). For quantitative real-time PCR, mRNA was reverse-transcribed to complementary DNA (cDNA) using the iScript cDNA Synthesis Kit (Bio-Rad, Hercules, CA), and qRT-PCR was performed on a StepOnePlus machine (Applied Biosystems, Carlsbad, CA) under default thermal cycling conditions. The 2−ΔΔCT method was used for data analysis, and mRNA levels were normalized to β-actin expression for each sample. The primer sequences are presented in **Table 1**.

**Table 1:** Primer information table.

Gene name	Forward	Reverse
Fasn	GGAGGTGGTGATAGCCGGTAT	TGGGTAATCCATAGAGCCCAG
Acc1	ATGGGCGGAATGGTCTCTTTC	TGGGGACCTTGTCTTCATCAT
Srebp1c	TGACCCGGCTATTCCGTGA	CTGGGCTGAGCAATACAGTTC
Chrebp	AGATGGAGAACCGACGTATCA	ACTGAGCGTGCTGACAAGTC
Ucp1	AGGCTTCCAGTACCATTAGGT	CTGAGTGAGGCAAAGCTGATTT
PGC-1α	TATGGAGTGACATAGAGTGTGCT	CCACTTCAATCCACCCAGAAAG
β-actin	GGCTGTATTCCCCTCCATCG	CCAGTTGGTAACAATGCCATGT

### 2.4 Blood glucose and insulin analysis

Blood glucose was measured using a Sinocare glucose meter (GA-3, China). Plasma insulin levels were determined using an ultrasensitive mouse insulin ELISA kit (ab277390, USA) according to the manufacturer’s instructions.

### 2.5 Body temperature measurement system

Mouse body temperature was measured using a small animal temperature analyzer. Specifically, temperature probes were placed near the oral cavity and interscapular brown adipose tissue (BAT), with readings taken for at least 10 seconds to allow stabilization. Two types of temperature conditions were used: first, room temperature (RT) conditions without any manipulation; and second, cold-induced conditions where mice were acclimated at 4°C for 6 hours before temperature measurement.

### 2.6 Measurement of inflammatory markers

Inflammatory markers were measured from mouse serum samples. Plasma IL-6, IL-1β, and TNF-α levels were determined using enzyme-linked immunosorbent assays (ELISA) according to the manufacturer’s instructions.

### 2.7 HE staining and Oil Red O staining

Fresh tissues were fixed in 4% paraformaldehyde for 24 hours, then sectioned at 5 μm thickness. After hydration in distilled water, sections were stained with hematoxylin for a few minutes, differentiated in acid water and ammonia water, rinsed in running water for 1 hour, and dehydrated in 70% and 90% alcohol for 10 minutes each. Sections were then counterstained with eosin for 2-3 minutes, cleared in xylene, and mounted for microscopic observation. For Oil Red O staining, sections were washed in 60% isopropanol, stained with 0.5% Oil Red O solution for 10 seconds, differentiated again in 60% isopropanol, and mounted for microscopy.

### 2.8 Statistical analysis

Results are presented as mean ± SE. Statistical analyses were performed using GraphPad Prism v8.4 (San Diego, CA). Comparisons between two groups were made using two-tailed Student’s t-tests, while one-way or two-way ANOVA was used for comparisons among three or more groups. Unless otherwise specified, P < 0.05 was considered statistically significant.

## 3 Results

### 3.1 Time-restricted feeding improves insulin sensitivity and reduces serum inflammation in mice on a normal diet

**Figure 1:**
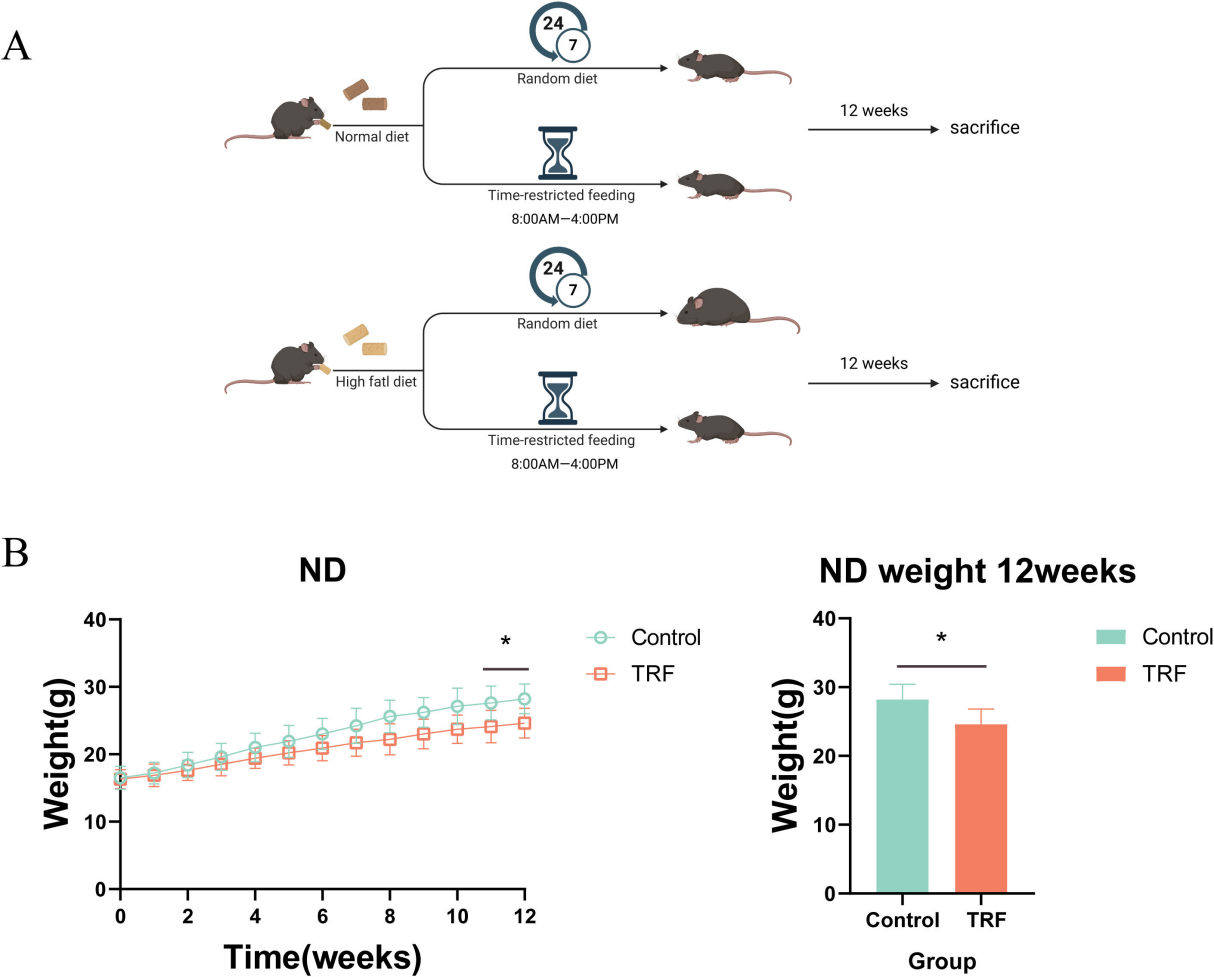
Time-restricted feeding (TRF) experimental design for mice on normal and high-fat diets. **(A)** Schematic representation of the TRF regimen for normal diet (ND) and high-fat diet (HFD) groups. Control represents the control group, TRF represents the time-restricted feeding group, with “normal diet” indicating the standard chow diet and “HFD diet” indicating the high-fat diet. **(B)** Effect of TRF on body weight regulation in mice on a normal diet, with a 12-week observation period, n=6. The bar graph shows the body weight differences at the 12-week endpoint, n=6. *P<0.05, **P<0.01, ***P<0.001.

**Figure 2:**
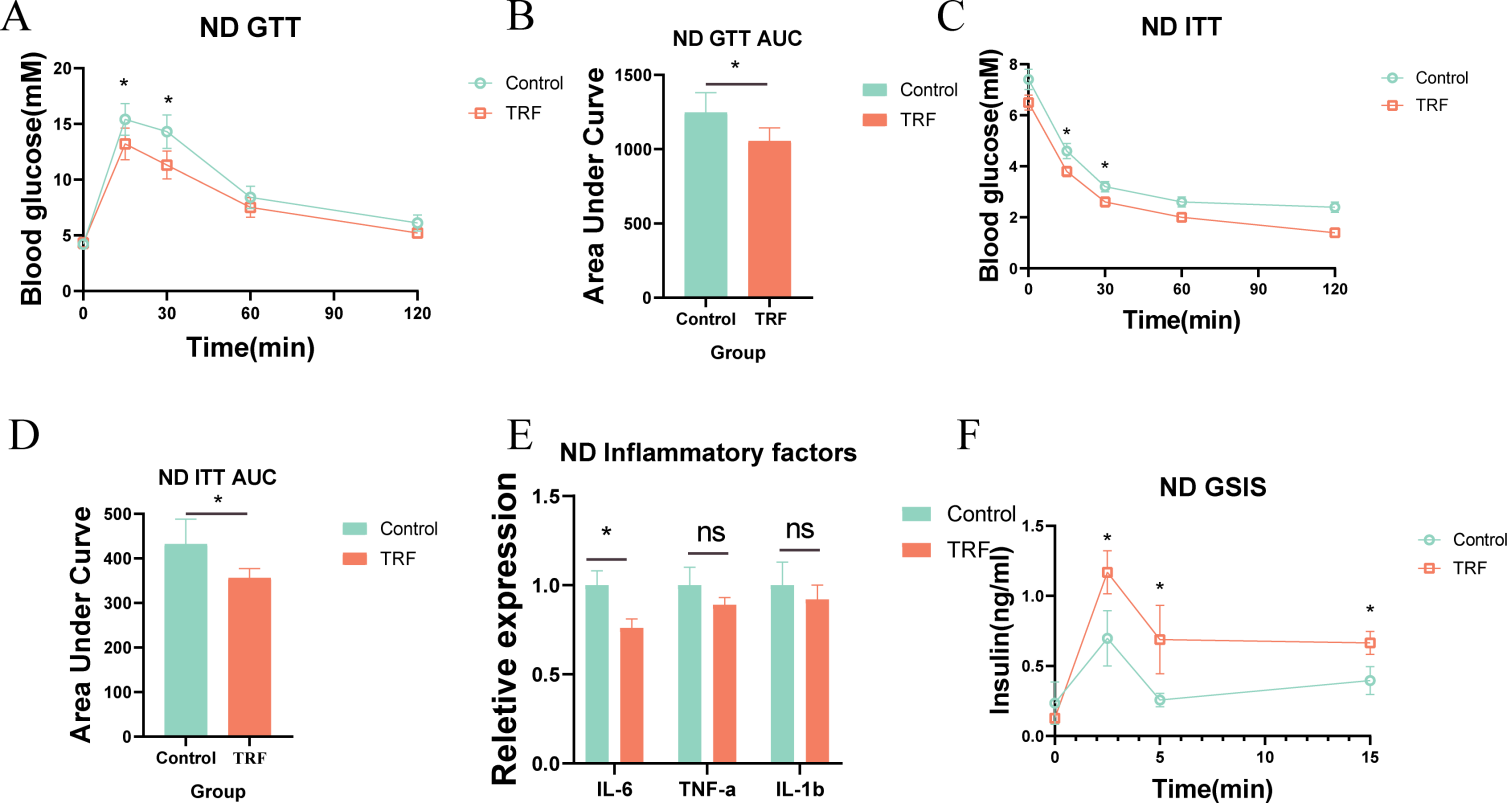
TRF improves glucose tolerance and insulin sensitivity in mice on a normal diet. **(A)** Glucose tolerance in TRF mice on a normal diet and **(B)** comparison of area under the curve (AUC). **(C)** Insulin sensitivity in TRF mice on a normal diet and **(D)** comparison of AUC. **(E)** TRF reduces serum inflammatory cytokines (IL-6, IL-1β, TNF-α) in mice on a normal diet. **(F)** TRF enhances glucose-stimulated insulin secretion in mice on a normal diet.A-D, n=6. E-F, n=4. *P<0.05, **P<0.01, ***P<0.001.

To investigate the effects of time-restricted feeding (TRF) on metabolism and inflammation, we first used mice on a normal diet as the study subjects. The experimental group underwent a 16-hour fasting period with unrestricted access to water (8:00 AM to 12:00 AM) and an 8-hour feeding window, while the control group had free access to food and water. The observation period lasted 12 weeks (**Figure 1A**). Results showed that during the 12-week observation, there was no significant difference in body weight between the TRF group and the control group initially. However, by weeks 11 and 12, the TRF group showed a significantly lower body weight compared to the control group, with a reduction of approximately 15% (**Figure 1B**). This suggests that TRF (16:8) can reduce body weight in mice on a normal diet. To further evaluate the effects of TRF on glucose metabolism, we conducted glucose tolerance tests (GTT) and insulin sensitivity tests (ITT). The results revealed that TRF mice exhibited significantly improved glucose tolerance compared to the control group, as evidenced by a lower area under the glucose tolerance curve. Moreover, TRF enhanced insulin sensitivity, particularly at the 15- and 30-minute time points during the insulin test (**Figures 2A–D**). These findings indicate that TRF improves both glucose tolerance and insulin sensitivity in mice on a normal diet. The underlying mechanism for this improvement in glucose tolerance is unclear, but we hypothesize that TRF may activate pancreatic β-cells to release insulin rapidly upon glucose stimulation. To test this, we performed glucose-stimulated insulin secretion assays. The results showed that, 2.5 minutes after glucose injection, TRF mice exhibited a significantly higher insulin release compared to the control group. This enhanced insulin secretion persisted at 5 and 15 minutes post-injection (**Figure 2F**). This suggests that TRF positively regulates insulin secretion by pancreatic β-cells, possibly by increasing the number or function of β-cells. Additionally, to assess the impact of TRF on inflammation, we measured serum levels of IL-6, IL-1β, and TNF-α. The results showed that TRF significantly reduced serum IL-6 levels, while no significant changes were observed for IL-1β and TNF-α, which may be due to the relatively low baseline inflammation in mice on a normal diet (**Figure 2E**). These findings suggest that TRF (16:8) improves insulin sensitivity and mildly reduces serum inflammation in normal-diet mice.

### 3.2 Time-restricted feeding improves insulin sensitivity, reduces inflammation, and exhibits anti-obesity effects in mice on a high-fat diet

**Figure 3:**
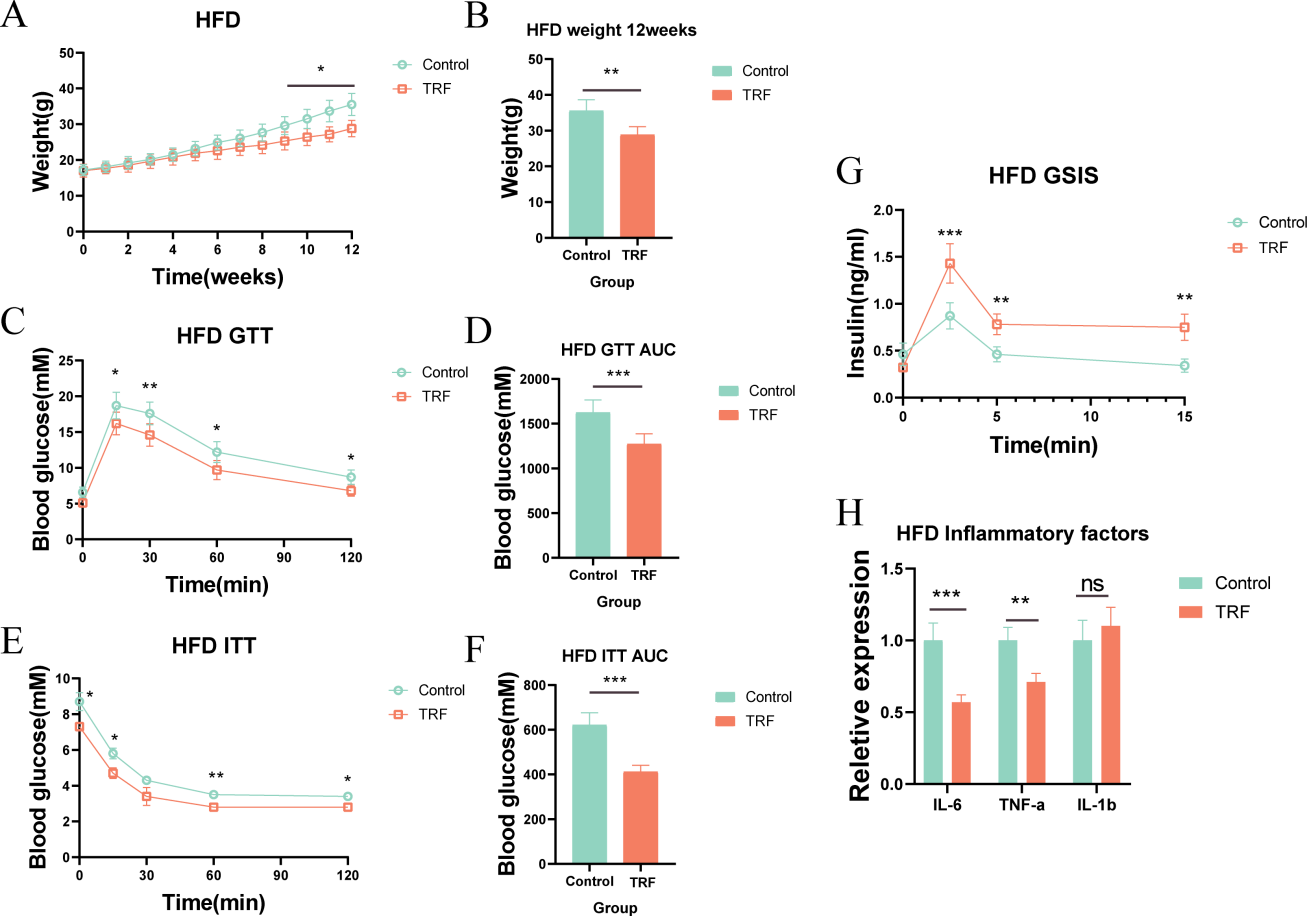
TRF improves glucose tolerance and insulin sensitivity in mice on a high-fat diet (HFD). **(A)** Body weight regulation in HFD mice under TRF during a 12-week observation period. **(B)** Body weight differences at the 12-week endpoint for HFD mice. **(C)** Glucose tolerance in TRF HFD mice and **(D)** comparison of AUC. **(E)** Insulin sensitivity in TRF HFD mice and **(F)** comparison of AUC. **(G)** TRF reduces serum inflammatory cytokines (IL-6, IL-1β, TNF-α) in HFD mice. **(H)** TRF enhances glucose-stimulated insulin secretion in HFD mice.A-F, n=6. G-H, n=4. *P<0.05, **P<0.01, ***P<0.001.

Since TRF improved insulin sensitivity and reduced serum inflammation in normal-diet mice, we further explored its effects on metabolic syndrome in pathological states by using mice on a high-fat diet (HFD). We designed an experiment similar to that for the normal-diet mice, but replaced the standard diet with a high-fat diet (**Figure 1A**). Over the 12-week period, we found that TRF significantly slowed weight gain in HFD-fed mice compared to the control group, with the first significant difference appearing in week 9, and the difference becoming more pronounced by week 12 (**Figures 3A, B**). To assess the impact of TRF on glucose metabolism in HFD-fed mice, we performed GTT and ITT. TRF improved glucose tolerance, with the most significant effect observed at 30 minutes post-glucose injection, and a sustained effect through 120 minutes. Insulin sensitivity was also improved, with TRF mice showing significantly lower blood glucose levels 15 minutes after insulin injection compared to the control group, and this difference persisted through 120 minutes (**Figures 3C–F**). Additionally, glucose-stimulated insulin secretion assays showed that TRF enhanced insulin release in HFD-fed mice, with a significant increase in insulin secretion 2.5 minutes after glucose injection, and this effect persisted through 15 minutes (**Figure 3G**). These results suggest that TRF enhances the responsiveness of pancreatic β-cells to glucose, possibly by increasing their number or insulin secretion capacity. To investigate the effect of TRF on inflammation in HFD-fed mice, we measured serum levels of IL-6, IL-1β, and TNF-α. TRF significantly reduced serum levels of IL-6 and TNF-α, but no significant change was observed for IL-1β (**Figure 3H**). These findings suggest that TRF reduces inflammation in HFD-fed mice, alongside improving insulin sensitivity and attenuating obesity.

### 3.3 Time-restricted feeding reduces hepatic lipid accumulation by downregulating lipogenic genes

**Figure 4:**
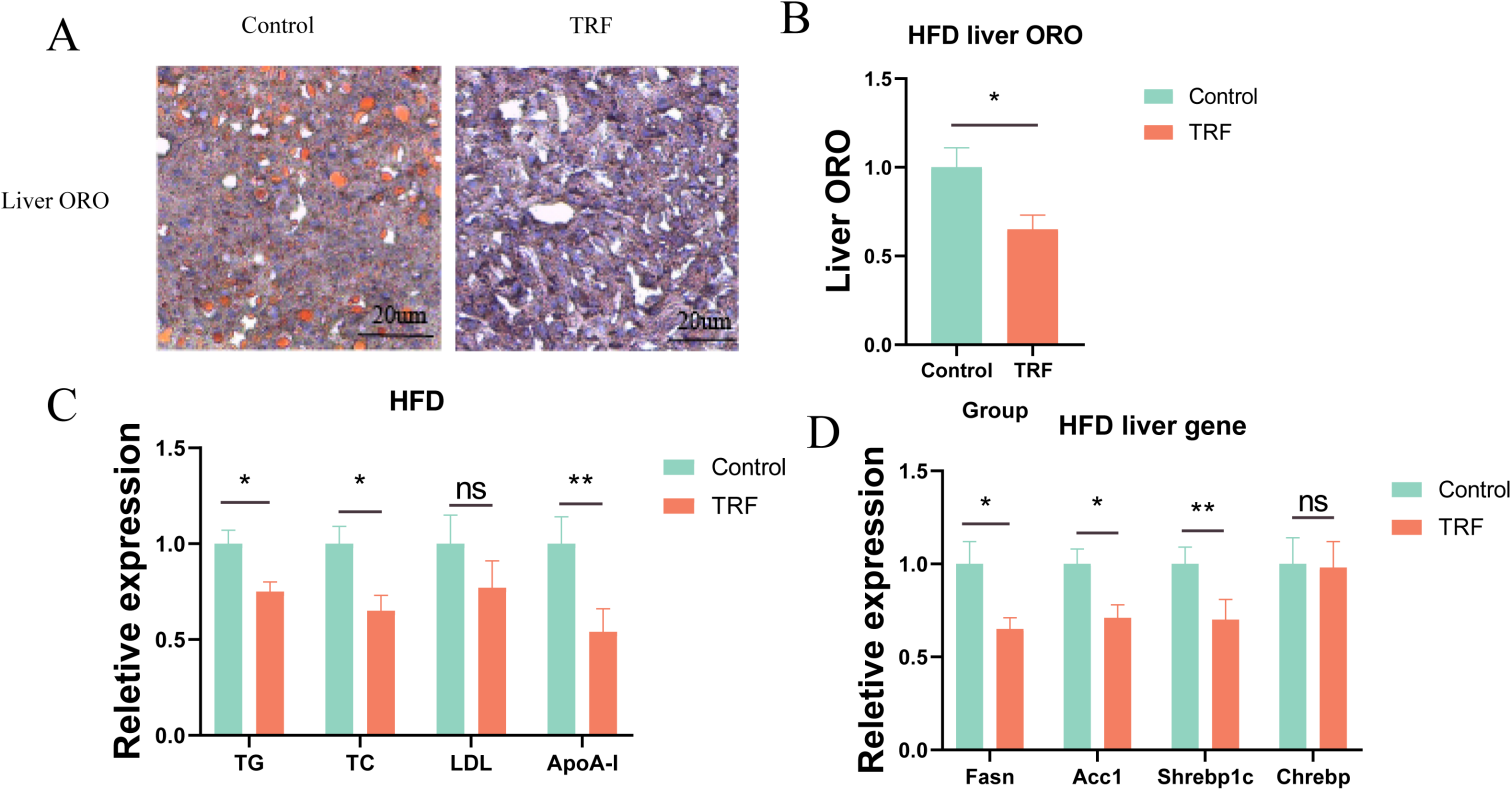
TRF improves lipid metabolism in HFD mice. **(A)** Oil Red O staining of liver sections from HFD mice following TRF. **(B)** Quantification of lipid content from liver sections stained with Oil Red O. **(C)** Effect of TRF on serum lipid levels (TG, TC, LDL, and ApoA1) in HFD mice. **(D)** TRF reduces the expression of hepatic lipogenic genes (Fasn, Acc1, Srebp1c, and Chrebp) in HFD mice.n=4. TG, triglycerides; TC, total cholesterol; LDL, low-density lipoprotein; ApoA1, apolipoprotein A1. *P<0.05, **P<0.01, ***P<0.001.

We further explored whether TRF improves hepatic lipid accumulation. Using Oil Red O staining, we examined lipid accumulation in the livers of HFD-fed mice. Results showed that sustained HFD feeding induced substantial lipid accumulation and vacuolar degeneration in the liver, while TRF significantly reduced both hepatic lipid accumulation and vacuolar degeneration, with a reduction of approximately 30% in lipid content compared to the control group (**Figures 4A, B**). These results suggest that TRF can reverse hepatic steatosis induced by a high-fat diet. To further understand the mechanism, we measured serum lipid levels, including triglycerides (TG), total cholesterol (TC), low-density lipoprotein (LDL), and apolipoprotein A1 (ApoA-1). TRF significantly reduced serum TG, TC, and ApoA-1 levels but had no effect on LDL levels (**Figure 4C**), indicating that TRF lowers serum lipid levels, which may contribute to its protective effect against hepatic steatosis. To investigate whether TRF reduces hepatic lipid accumulation by downregulating lipid synthesis, we measured the expression of lipogenic genes (Fasn, Acc1, Srebp1c, and Chrebp). TRF significantly downregulated the expression of Fasn and Acc1, both of which are closely related to lipid synthesis, as well as Srebp1c, a key gene associated with non-alcoholic fatty liver disease (**Figure 4D**). These findings suggest that TRF ameliorates hepatic steatosis by reducing the expression of lipogenic genes.

### 3.4 Time-restricted feeding regulates brown adipose tissue thermogenesis and reduces white adipose tissue accumulation

**Figure 5:**
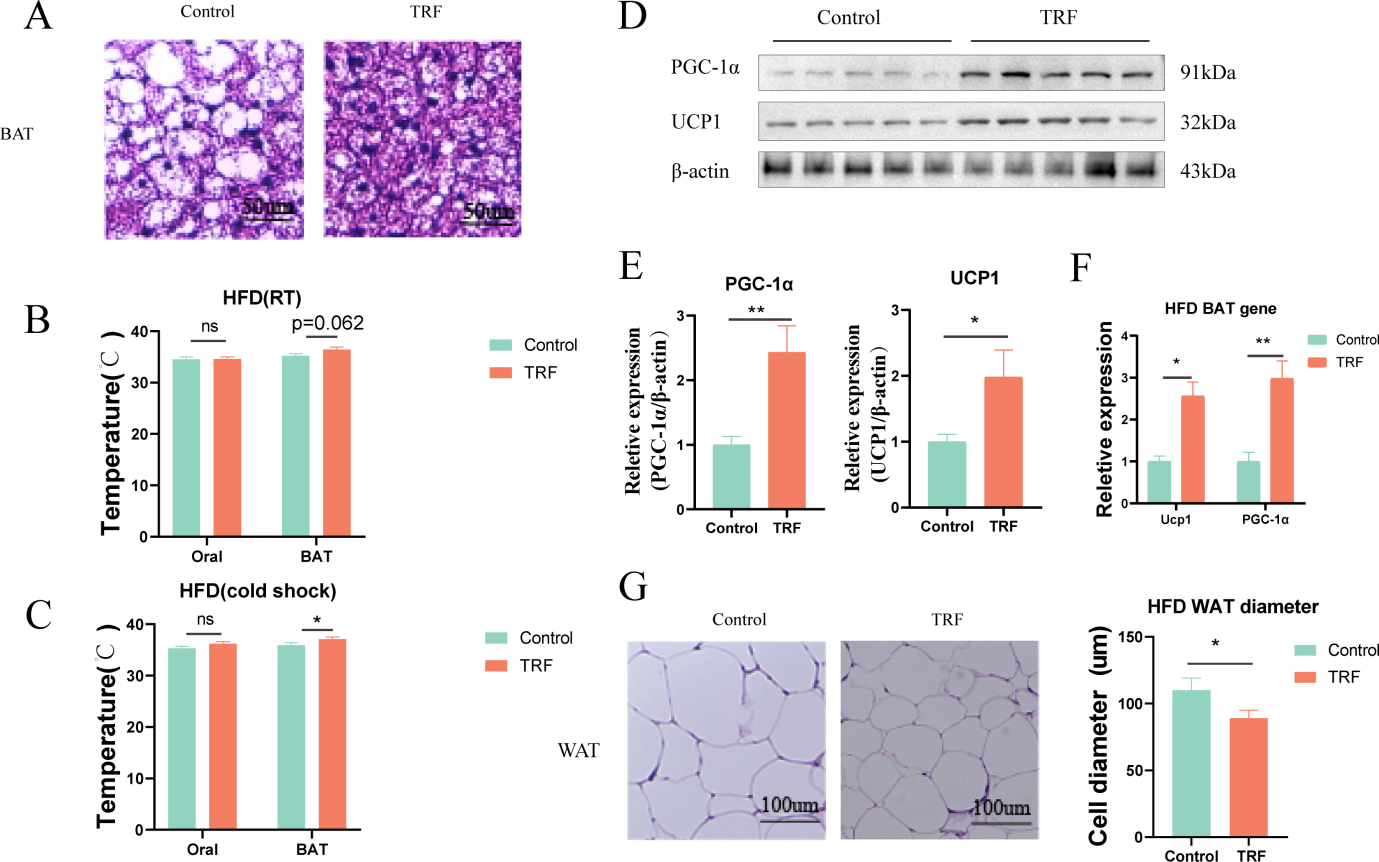
TRF enhances brown adipose tissue (BAT) thermogenesis in HFD mice. **(A)** HE staining of frozen BAT sections from HFD mice after TRF. **(B)** Effect of TRF on oral and interscapular BAT temperature at room temperature in HFD mice. **(C)** Effect of TRF on oral and interscapular BAT temperature under cold-induced conditions in HFD mice. **(D–F)** TRF increases the expression of thermogenic genes and protein(Ucp1 and PGC-1α) in BAT of HFD mice. **(G)** TRF reduces white adipose tissue (WAT) cell size in HFD mice. The bar graph shows differences in adipocyte diameter. n=4-5. *P<0.05, **P<0.01, ***P<0.001.

Fasting has been found to improve glucose and lipid metabolism in mice fed a high-fat diet (HFD), particularly by reducing hepatic steatosis. Previous studies have shown that the effects of fasting on lipid accumulation are not limited to reducing lipid intake but also include increasing energy expenditure. Therefore, we further investigated the thermogenic effects of brown adipose tissue (BAT), which is a key organ involved in systemic energy metabolism.First, we performed histological staining of BAT from HFD-fed mice to observe changes in BAT size and the whitening effect of BAT under HFD challenge. The results showed that in the control group, BAT exhibited typical whitening under prolonged HFD feeding, characterized by the appearance of large lipid vacuoles, resembling those of white adipose tissue (**Figure 5A**). In contrast, fasting reversed this whitening phenomenon in the fasting group, where BAT remained denser, without large lipid vacuoles. This indicates that fasting has a protective effect against the whitening of BAT. However, the protective mechanism of fasting on BAT remains unclear.Literature suggests that BAT, as a thermogenic organ, has a heat-generating capacity closely related to its brown phenotype, and increased thermogenesis can result in elevated body temperature. To investigate further, we measured the oral and interscapular BAT temperatures in mice using a small animal temperature monitoring device. The results indicated that, in normal diet-fed mice, fasting had minimal impact on BAT thermogenesis under either room temperature (RT) or cold-induced (cold shock) conditions. However, in HFD-fed mice, fasting enhanced thermogenesis in the interscapular BAT region following cold shock, though no significant differences in thermogenesis were observed between the groups under RT conditions (**Figures 5B, C**).To verify these phenotypic observations, we measured the expression levels of key thermogenic genes and proteins (UCP1 and PGC-1α) in BAT. Our results demonstrated that time-restricted fasting increased the expression of both UCP1 and PGC-1α at the gene and protein levels in BAT (**Figures 5D–F**), which could explain the observed rise in BAT temperature in the fasting group. These findings suggest that fasting can regulate BAT thermogenesis, potentially contributing to its therapeutic effects on metabolic syndrome.Additionally, epididymal white adipose tissue (WAT), a representative of white fat, was used to assess fasting’s effects on lipid accumulation. We performed histological staining of epididymal WAT from HFD-fed mice and measured adipocyte diameter. The results showed that fasting reduced adipocyte diameter in epididymal WAT (**Figure 5G**). We hypothesize that this change may be due to a combination of reduced serum lipid levels and increased BAT thermogenesis induced by fasting.

**Figure 6:**
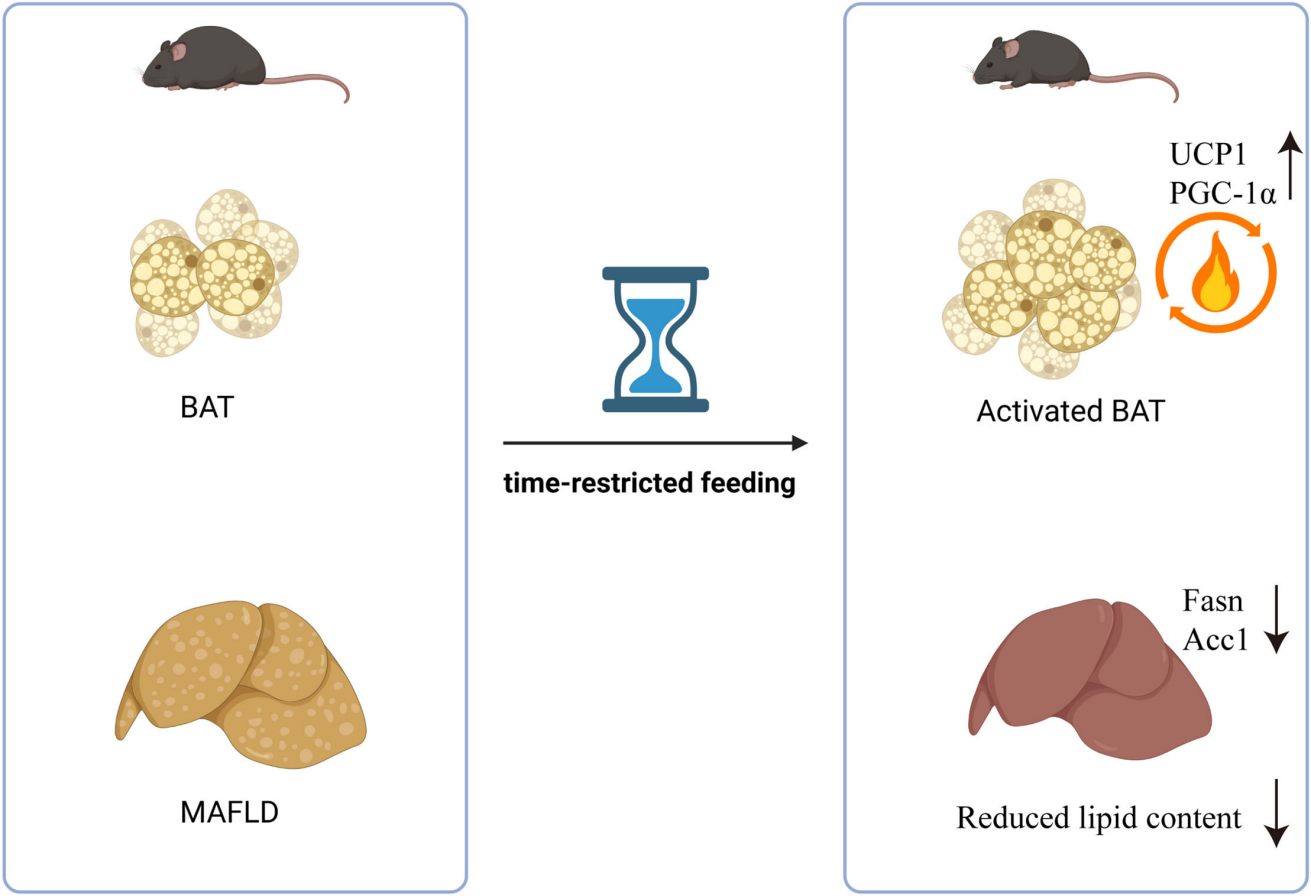
Conceptual diagram illustrating the metabolic benefits of TRF in mice.

These results provide insight into the mechanisms by which fasting influences fat thermogenesis and lipid accumulation, offering a potential explanation for its beneficial effects in the treatment of metabolic syndrome (**Figure 6**).

## 4 Discussion

Preclinical and clinical studies have shown that intermittent fasting has broad benefits for many diseases, including obesity, type 2 diabetes, and hypertension, as well as improving cardiovascular risk factors (25). In our study, we found that time-restricted fasting (16 + 8 mode) can improve metabolic syndrome by activating brown fat thermogenesis and reducing inflammatory markers.

In this study, we used mice fed a normal diet as a surrogate for the general population. Through 12 weeks of time-restricted fasting, we found that time-restricted fasting can improve insulin sensitivity and reduce serum inflammation in mice on a normal diet, but the change in body weight is very weak. Our findings are different from (26), who found that time-restricted fasting can reduce the body weight of mice on a normal diet. But the fasting mode they adopted is also different from ours. Li et al. adopted fasting every other day, and they improved metabolic syndrome by affecting intestinal microbial metabolism. The weight phenotype of mice on a normal diet was not found in the fasting mode we adopted. However, our findings on improving insulin sensitivity and serum inflammation are consistent with Li (26). This also further suggests that the improvement of metabolic syndrome by the 16 + 8 fasting mode may be related to the abundance and metabolism of intestinal microorganisms, although this item was not involved in our study. In addition, related studies have also suggested that time-restricted fasting has inconsistent effects on weight loss in different diets (high-fat or normal) (27). This may be because a high-fat diet can amplify the weight phenotype. As for the reason why time-restricted fasting improves insulin resistance, we believe that time-restricted fasting may have an activating effect on insulin receptor targets in skeletal muscle and related organs, which requires further study. Recent studies have also explained the beneficial effects of time-restricted fasting on insulin resistance, believing that time-restricted fasting may regulate phenotypic changes caused by fat metabolism through the PPAR pathway (28). Zhang et al. believe that time-restricted fasting reduces insulin resistance by activating the SIRT3 pathway (29). In this study, we are more convinced that PPAR activation activates insulin resistance caused by fat metabolism, so we observed changes in adipocyte size. Although we did not detect the protein level of PPAR, the level of UCP1 thermogenic protein similar to PPAR was found to be increased in our study.

We further used high-fat diet mice as a substitute for obese or Western diet people. We found that time-restricted fasting not only reduced the weight of high-fat diet mice, but also improved glucose tolerance and insulin resistance in mice. We further found that time-restricted fasting reduced low-grade inflammation in high-fat diet mice and improved liver fat accumulation and white adipocyte size. Our results are consistent with those of Reinisch (30) and other studies. As for the improvement of liver fat accumulation, related studies (31) believe that it is mediated by cup apoptosis, although we did not explore this point in depth. But importantly, we found that fasting can increase the brown fat heat production in mice. At room temperature, the brown fat temperature of time-restricted fasting mice was slightly higher than that of the control group, and after cold induction, this phenomenon was very obvious. This change may be related to the activation of brown fat heat production genes (Ucp1 and PGC-1α). In the study of Li (26), it was found that time-restricted fasting may promote the browning of white fat by reshaping intestinal microorganisms, further improving the obese phenotype. As a new explanation, this provides a new idea for time-restricted fasting to improve metabolic syndrome. Other studies have shown that intermittent fasting promotes fat thermogenesis and metabolic homeostasis through VEGF-mediated alternative activation of macrophages (32). However, in further studies, we found that time-restricted fasting can also reduce the expression level of hepatic lipid production genes, which may be beneficial for the treatment of non-alcoholic fatty liver disease. In short, our findings provide another new perspective on time-restricted fasting, which can increase brown fat thermogenesis. In addition, we also observed that after time-restricted fasting, the insulin secretion of mice after glucose stimulation increased significantly, which may be related to the increase in the number of pancreatic β cells or the enhancement of secretory function. This phenomenon has been reported by researchers such as Matthew (33). In addition, relevant researchers (34) also proposed that time-restricted fasting can alleviate metabolic syndrome by regulating bile acid, but this direction was not explored in this study. Although time-restricted fasting has been used clinically as a powerful and simple dietary regulation method, it can prolong the lifespan and health lifespan of model organisms and improve various diseases (35). However, it is necessary to clarify the internal preparations involved in order to recommend corresponding benefits for treatment. Nevertheless, our study did not observe or detect the number of calories consumed by mice with time-restricted fasting every day. This requires further study in our subsequent studies to further study whether the weight loss is the result of the combined effect of reduced calorie intake and energy expenditure while controlling calorie intake. Although this study suggests that the 16 + 8 fasting mode can improve metabolic syndrome by activating brown fat thermogenesis and reducing inflammatory markers, these studies are limited to animals and these results have not been observed in humans. We expect that subsequent studies will make up for this limitation.

In conclusion, in this study, we found that time-restricted fasting can activate brown fat thermogenesis and reduce inflammatory markers, and this effect will be beneficial to improve hepatic steatosis and reduce white fat accumulation.

## 5 Conclusion

In conclusion, we found that TRF improves metabolic health by enhancing BAT thermogenesis and reducing inflammatory markers, offering potential benefits for managing hepatic steatosis and reducing white adipose tissue accumulation.

## Data Availability

The original contributions presented in the study are included in the article/supplementary material. Further inquiries can be directed to the corresponding authors.
